# Humic Acid/Multi-Walled Carbon Nanotube Composites: Influence of Ultrasonic Treatment Duration on Structure, Physicochemical Properties, and Phenol Adsorption

**DOI:** 10.3390/ma19132833

**Published:** 2026-07-02

**Authors:** Alma Khassenovna Zhakina, Oxana Vasilievna Arnt, Yevgeniy Petrovich Vassilets, Almat Maulenuly Zhakin, Abylaikhan N. Bolatbay, Zainulla Muldakhmetov

**Affiliations:** 1 LLP “Institute of Organic Synthesis and Coal Chemistry of the Republic of Kazakhstan”, Karaganda 100008, Kazakhstan; oxana230590@mail.ru (O.V.A.); vassilets88@mail.ru (Y.P.V.); zhakin-almat@mail.ru (A.M.Z.); iosu.rk@mail.ru (Z.M.); 2 Department of Chemical Technology and Ecology, Faculty of Metallurgy and Mechanical Engineering, Karaganda Industrial University, Temirtau 101400, Kazakhstan; 3 Chemistry Faculty, Karaganda Buketov University, Karaganda 100024, Kazakhstan; abylai_bolatbai@mail.ru

**Keywords:** composite materials, humic acids, multi-walled carbon nanotubes, ultrasonic treatment, phenol adsorption

## Abstract

Composite materials based on humic acids and multi-walled carbon nanotubes were synthesized using ultrasonic-enhanced co-precipitation. The effect of ultrasonic treatment duration on the structure and adsorption properties of the composite materials with respect to phenol was studied. The structural and functional characteristics of the materials were investigated using elemental analysis, FTIR spectroscopy, SEM, TGA/DTA, and determination of the content of oxygen-containing functional groups. It was found that the Σ(COOH+OH) values for the studied composites are in the range of 3.00–4.45 mmol/g. The highest value of this indicator was observed for the HA:MWCNTs-20 (US = 30 min) composite. The results of physicochemical studies show that the ultrasonic treatment duration has a significant effect on the morphological, functional, and thermal characteristics of the composites. Adsorption properties were studied in the phenol concentration range of 0.5–15 mg/dm^3^. It was shown that the HA:MWCNTs-20 (US = 30 min) composite exhibited the highest adsorption capacity for phenol among the studied samples. Analysis of adsorption isotherms revealed that the experimental data were most satisfactorily described by the Langmuir model (r = 0.996–0.999), while the kinetic data were best described by the pseudo-second-order model. These results demonstrate the potential of HA:MWCNTs composites as sorption materials for extracting phenol from aqueous solutions.

## 1. Introduction

In recent years, considerable attention has been devoted to the development of functional carbon-containing materials for solving environmental problems associated with the purification of natural and wastewater from organic contaminants. Phenolic compounds are among the most hazardous pollutants due to their widespread occurrence in wastewater generated by the petrochemical, coke-chemical, metallurgical, and coal-processing industries. Their high toxicity, resistance to biodegradation, and ability to exert adverse effects on aquatic ecosystems and human health even at low concentrations necessitate the development of effective methods for their removal from aqueous media [1,2,3]. In this regard, the development of new sorption materials combining high efficiency in the removal of phenolic pollutants, availability of raw materials, and environmental safety is of considerable scientific and practical interest.

Among the promising materials for addressing this problem, carbon nanomaterials occupy a special place owing to their high specific surface area, excellent chemical and thermal stability, and broad possibilities for targeted surface modification. Multi-walled carbon nanotubes (MWCNTs) are among the most extensively studied representatives of this class. Due to their structure, MWCNTs are capable of efficiently interacting with aromatic organic compounds through hydrophobic and π–π interactions [4,5,6,7,8,9,10,11,12].

A promising approach for improving the functional properties of carbon materials is their modification with naturally occurring high-molecular-weight compounds. In this respect, humic acids (HA) are of particular interest. Humic acids are natural polyfunctional macromolecular systems containing carboxyl, phenolic, and hydroxyl groups. The presence of a wide range of functional centers determines the ability of HA to participate in complexation, ion-exchange, and adsorption processes involving various pollutants [13,14,15,16,17,18,19]. In addition, the use of humic acids isolated from carbon-containing raw materials contributes to the expansion of integrated natural resource utilization and the production of materials with high added value.

Published studies indicate that the combination of humic acids with carbon nanomaterials makes it possible to obtain materials with improved structural, functional, and sorption characteristics [20,21,22,23,24,25,26]. However, most studies have focused on the removal of heavy metal ions and dyes, whereas the structural features of HA composites and their interactions with phenolic compounds remain insufficiently investigated. Furthermore, information concerning the influence of ultrasonic treatment on the formation of the composite structure, distribution of oxygen-containing functional groups, thermal behavior, and adsorption properties remains limited. To more clearly identify the existing research gap and the specific contribution of the present study, Table 1 summarizes representative studies on humic acid/carbon nanomaterial-based composites and compares them with the approach adopted in this work.

As shown in Table 1, previous studies have mainly focused on the preparation of composite adsorbents and their application for the removal of heavy metal ions and organic dyes. In contrast, the influence of ultrasonic treatment duration on the physicochemical characteristics of HA composites and its relationship with phenol adsorption has received limited attention.

Therefore, the present study aims to address this research gap by comprehensively investigating the influence of ultrasonic treatment duration on the structure, functional-group accessibility, morphology, thermal stability, and adsorption performance of HA composites prepared with different MWCNT contents. The novelty of this work lies in establishing the relationship between synthesis conditions, physicochemical characteristics, and phenol adsorption performance, as well as in identifying the optimal composite composition and ultrasonic treatment conditions for obtaining an efficient sorbent for phenol removal from aqueous solutions.

## 2. Materials and Methods

### 2.1. Materials

Humic acids (HA) isolated from oxidized coal waste of the Shubarkol deposit (Karaganda, Kazakhstan) were used as starting components for the preparation of composite materials based on humic acids and multi-walled carbon nanotubes [22]. The HA were used in the acid form.

Multi-walled carbon nanotubes (MWCNTs) supplied by Sigma-Aldrich (St. Louis, MO, USA, 755125-1G) were used as the modifying component. The characteristics of the MWCNTs were as follows: carbon content greater than 98%, outer diameter 6.0–13.0 nm (average outer diameter 8.7–10 nm), inner diameter 2.0–6.0 nm, length 2.5–20 μm (average length approximately 10 μm), and BET specific surface area of 216 m^2^/g.

The MWCNTs were used without preliminary chemical functionalization. To improve the degree of dispersion, the nanotubes were preliminarily subjected to ultrasonic treatment for 30 min. The resulting dispersions were directly used for the synthesis of HA:MWCNTs carbon-containing materials by ultrasound-assisted co-precipitation.

Distilled water was used for the preparation of all solutions. The pH was adjusted using analytical-grade 0.1 M NaOH and 0.1 M HCl solutions. Adsorption studies were carried out using freshly prepared aqueous phenol solutions with initial concentrations ranging from 0.5 to 15 mg/dm^3^.

### 2.2. Synthesis of Composite Materials Based on Humic Acids and MWCNTs

Composite materials based on humic acids (HA) and multi-walled carbon nanotubes (MWCNTs) were prepared by an ultrasound-assisted co-precipitation method. The synthesis was carried out at three HA:MWCNTs mass ratios of 90:10, 80:20, and 70:30 (wt.%), with the total composite mass maintained at 5 g.

At the first stage, a weighed portion of MWCNTs was dispersed in 100 cm^3^ of distilled water with preliminary adjustment of the suspension pH to 9.0 using a NaOH solution. Dispersion was performed using an IL 100-6/2 (St. Petersburg, Russia) ultrasonic generator equipped with a magnetostrictive transducer operating at a frequency of 22 kHz and an output power of 60 W, fitted with a cylindrical waveguide. Ultrasonic treatment durations of 15, 30, and 60 min were selected to evaluate the influence of short-, intermediate-, and prolonged ultrasonic exposure on the physicochemical characteristics and adsorption properties of the synthesized composites.

Ultrasonic treatment (US) was carried out in a pulsed mode with temperature control of the reaction medium (not exceeding 35 °C), achieved by placing the reaction vessel in a cooled water bath.

The resulting MWCNTs dispersion was slowly introduced into an aqueous HA solution under intensive mechanical stirring while maintaining the pH of the reaction system at 9.0. This mixing regime ensured a more uniform distribution of nanotubes throughout the system volume, reduced the probability of local aggregation, and promoted effective interaction between the functional groups of humic acid and the MWCNTs surface.

After completion of mixing, the reaction system was additionally subjected to ultrasonic treatment to intensify interfacial interactions between the components.

At the next stage, the system was acidified with HCl solution to pH 2.0–2.5 and maintained under continuous stirring for 30 min.

The resulting precipitate was allowed to age at room temperature for 24 h, after which it was separated by filtration, washed with distilled water until a neutral reaction medium (pH 6.5–7.0) was achieved, and dried at 60 °C to constant weight. The dried samples were ground in an agate mortar to obtain a homogeneous powder.

The product yield was calculated as the ratio of the mass of the obtained composite material to the total mass of the initial components.

The resulting composite materials were designated as HA:MWCNTs-10 (90:10), HA:MWCNTs-20 (80:20), and HA:MWCNTs-30 (70:30), where the numerical index corresponds to the mass fraction of MWCNTs in the composite.

### 2.3. Investigation of the Adsorption Properties of Composite Materials

The adsorption properties of HA and the HA:MWCNT-20 (80:20) composite materials synthesized on its basis toward phenol were investigated under static conditions with continuous stirring of the system.

The experiments were carried out at a temperature of 25 °C using 0.1 g of sorbent and 100 cm^3^ of phenol solution with an initial concentration of 0.5–15 mg/dm^3^. To ensure uniform distribution of the sorbent and attainment of adsorption equilibrium, the system was continuously stirred for 24 h. The process was conducted in a closed system without solution flow.

After completion of the experiment, the solid phase was separated from the solution by filtration. The residual phenol concentration was determined in the resulting filtrate.

Quantitative determination of phenol was performed by a fluorimetric method using a Fluorat-02-2M liquid analyzer (Lumex Ltd., St. Petersburg, Russia) in accordance with [27]. The method is based on the extraction of phenols from the aqueous phase with butyl acetate, followed by re-extraction into an aqueous sodium hydroxide solution and measurement of their content by fluorescence intensity after acidification of the re-extract.

During the measurements, fluorescence excitation of phenolic compounds, registration of the analytical signal, and automatic calculation of phenol concentration using a calibration dependence incorporated into the analyzer software were performed.

Instrument calibration was carried out using a certified reference phenol solution in methanol [28]. The upper limit of the working range of quantitative determination for the equipment used was 20 mg/dm^3^.

Adsorption experiments were performed in triplicate, and the results are presented as average values. For a series of measurements (n = 3), the standard deviation was ±0.2 mg/g, indicating satisfactory reproducibility of the obtained results.

The adsorption properties of the obtained composite materials were evaluated using the adsorption capacity (mg/g) and phenol removal efficiency (%), calculated according to the following equations:

(1) $$A=\frac{{(C}_{0}-C_{eq})\cdot V}{m},$$

(2) $$R=\frac{C_{0}-C_{eq}}{C_{0}}\cdot 100\%,$$

where C_0_—the initial phenol concentration (mg dm^−3^), C_eq_—the equilibrium phenol concentration after adsorption (mg dm^−3^), V—the solution volume (dm^3^), and m—the sorbent mass (g).

Based on the experimental data, adsorption isotherms were constructed. The obtained sorption isotherms can theoretically be described using two equations.

(1) The Langmuir monolayer sorption model, which describes the sorption isotherm over the entire concentration range:

(3) $$A=\frac{A_{max}\cdot (K_{sorb}\cdot C_{eq})}{1+K_{sorb}\cdot C_{eq}},$$

or in linear form:

(4) $$\frac{C_{eq}}{A}=\frac{1}{K_{sorb}\cdot A_{max}}+\frac{1}{A_{max}}\cdot C_{eq},$$

where K_sorb_—the adsorption equilibrium constant (dm^3^ mg^−1^) and A_max_—the maximum adsorption capacity (mg g^−1^).

(2) The empirical Freundlich equation, which is commonly used to describe the intermediate region of a sorption isotherm:

(5) $$A=K_{Fr}\cdot C_{eq}^{1/n},$$

or in linear form:

(6) $$lgA=lgK_{Fr}+\frac{1}{n}\cdot lgC_{eq},$$

where K_Fr_ is the Freundlich constant representing the amount of adsorbate at a unit equilibrium concentration, and 1/n is a constant whose value is a proper fraction.

For additional evaluation of the energetic heterogeneity of the surface and adsorbate-adsorbent interactions, the experimental data were also analyzed using the Temkin model:

(7) $$q_{e}=BlnA+BlnC_{eq},$$

where B—the Temkin constant, A—the Temkin equilibrium constant; C_eq_—the equilibrium concentration of phenol.

To determine the pseudo-order, a graphical method was chosen using kinetic equations for reactions of integer order—first (8) and second (9):

(8) $$lnC=lnC_{0}-k_{1}t,$$

(9) $$\frac{1}{C}=k_{2}t+\frac{1}{C_{0}^{2}},$$

The contribution of intraparticle diffusion was evaluated using the Weber-Morris model (10):

(10) $$q_{t}=K_{id}t^{1/2}+C,$$

where K_id_—the intraparticle diffusion rate constant and C—a constant related to boundary-layer effects.

### 2.4. Physicochemical Characterization Methods of HA:MWCNTs Composite Materials

The composition of the initial compounds and the synthesized composite materials was characterized using a set of physicochemical analytical techniques.

Elemental Analysis. The elemental composition of the initial compounds and synthesized composite materials (C, H, O) was determined by elemental analysis using an Elementar Unicube automatic elemental analyzer (Elementar Analysensysteme GmbH, Langenselbold, Germany). The method is based on the classical Pregl-Dumas procedure for organic elemental analysis and includes the following stages: combustion (reduction), homogenization of the reaction products, separation, and subsequent detection. The analysis was performed using samples weighing 1–5 mg placed in tin capsules. The combustion column temperature was maintained at 1150 °C, while the reduction column temperature was 850 °C. For each sample, at least three parallel measurements were carried out, and the average mass fractions of the corresponding elements were calculated.

Determination of Oxygen-Containing Functional Groups. Quantitative determination of oxygen-containing functional groups in HA and the synthesized composite materials was carried out by the conductometric titration method [29,30]. The total content of oxygen-containing functional groups, Σ(COOH+OH), was determined by direct and back conductometric titration using 0.1 M NaOH and 0.1 M HCl solutions as titrants.

Electrical conductivity measurements were performed using an Anion-4100 laboratory conductometer (Research and Production Enterprise “Anion”, Moscow, Russia).

A sample weighing 0.1 g was dispersed in deionized water under continuous stirring until a stable conductivity value was achieved. Titration was carried out at 25 °C with continuous recording of the dependence of solution conductivity on the volume of titrant added, followed by construction of conductometric curves.

The equivalence point was determined from the inflection point of the conductometric curve corresponding to the neutralization of carboxyl and phenolic hydroxyl groups.

The total content of oxygen-containing functional groups was calculated from the volume of titrant consumed up to the equivalence point, taking into account the titrant concentration and sample mass, according to the following equation:

(11) $$\Sigma (COOH+OH)=\frac{C\cdot V}{m},$$

where C—the titrant concentration (mol/dm^3^), V—the volume of titrant consumed up to the equivalence point (dm^3^), and m—the sample mass (g). The results are expressed in mmol/g. All measurements were performed in triplicate. The relative standard deviation did not exceed 1%.

Fourier transform infrared spectroscopy. Functional groups in the samples were identified using infrared spectroscopy. FTIR spectra were recorded in the range of 400–4000 cm^−1^ with a spectral resolution of 4 cm^−1^ and by averaging 20 scans for each sample to improve signal-to-noise ratio using an FSM-1201 spectrometer (Infraspek, St. Petersburg, Russia). Samples were prepared using the standard method of pressing with KBr. The resulting spectra were processed and approximated using Fityk 1.3.1 software to identify characteristic functional groups in the samples.

Scanning Electron Microscopy (SEM). The surface morphology and distribution of multiwalled carbon nanotubes in a humic acid matrix were studied using scanning electron microscopy (SEM) on a MIRA 3 (TESCAN, Brno, Czech Republic) scanning electron microscope. The instrument is equipped with a system of detectors for various signal types, enabling the acquisition of images with high spatial resolution and detail necessary for analyzing the microstructure of the studied composites. Prior to analysis, the samples were coated with a thin gold layer to prevent surface charging. SEM analysis was performed at an accelerating voltage of 5,0 kV, an electron beam current of 86 pA, and a working distance of 3.3 mm. Images were recorded in secondary electron mode at magnifications up to ×50,000.

Differential thermal analysis (DTA). The thermal stability of the HA and composites was studied using simultaneous thermogravimetric and differential thermal analysis (DTA) on a PerkinElmer STA 6000 instrument (PerkinElmer, Inc., Shelton, CT, USA). Measurements were performed in an argon atmosphere at a gas flow rate of 50 mL/min over a temperature range from room temperature to 1000 °C with a heating rate of 10 °C/min. Corundum crucibles (Al_2_O_3_) were used as crucibles. The sample weight for each sample was selected individually.

## 3. Results and Discussion

### 3.1. Synthesis and Characterization of Composite Materials Based on Humic Acids and MWCNTs

**Figure 1:**
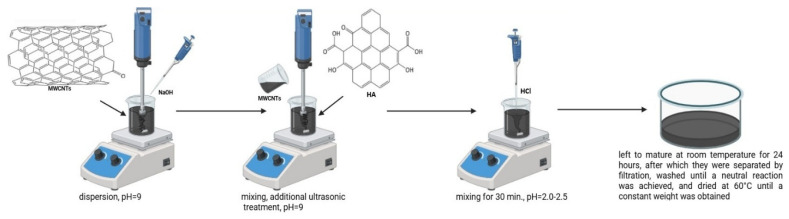
Graphical diagram of the synthesis of the HA:MWCNTs composites.

Figure 1 presents a schematic representation of the synthesis of the composite material.

The results of the chemical characterization of the synthesized composite materials are confirmed by elemental analysis, conductometric measurements, FTIR spectroscopy, thermogravimetric analysis, and scanning electron microscopy. Table 2 presents the results of elemental analysis, the total content of functional groups Σ(COOH+OH), and the yields of the initial HA and composite materials obtained at different component mass ratios.

Based on the comparative evaluation of the synthesized composites, the HA:MWCNTs-20 sample demonstrated the most favorable combination of product yield, oxygen-containing functional groups, elemental composition, and structural stability. Therefore, this sample was selected for detailed physicochemical characterization and adsorption studies.

The initial HA is characterized by a high oxygen content (48.31 wt.%), which is attributed to the presence of oxygen-containing functional groups, including carboxyl and phenolic groups [14,15], and is confirmed by the maximum value of Σ(COOH+OH) equal to 5.00 mmol/g.

Upon formation of composites with multi-walled carbon nanotubes, an increase in the carbon content (from 47.34 to 59.15 wt.%) and a corresponding decrease in oxygen content are observed, which may be associated with the contribution of the carbon phase of MWCNTs to the composition of the composites.

The data presented in Table 2 indicate that the values of Σ(COOH+OH) for the composites are in the range of 3.30–4.45 mmol/g, indicating the preservation of the functional groups of the humic component after ultrasound-assisted co-precipitation.

The highest value of Σ(COOH+OH) among the investigated composites was observed for HA:MWCNTs-20 (4.45 mmol/g), which may indicate greater accessibility of functional groups compared with the other investigated samples.

A comparative analysis shows that the transition from the initial HA (5.00 mmol/g) to the composites is accompanied by a decrease in Σ(COOH+OH): for HA:MWCNTs-10 to 3.83 mmol/g (24% decrease), for HA:MWCNTs-20 to 4.45 mmol/g (12% decrease), and for HA:MWCNTs-30 to 3.30 mmol/g (34% decrease).

The determination of Σ(COOH+OH) for each sample was performed in triplicate. Table 2 presents the average values, while the deviations between parallel measurements did not exceed 1%, indicating good reproducibility of the experimental data.

The absence of a monotonic decrease in this parameter with increasing MWCNTs content indicates the complex nature of interfacial interactions between the composite components.

The obtained results suggest that the decrease in Σ(COOH+OH) values is associated not only with changes in the total content of functional groups but also with a possible decrease in their accessibility due to interactions between humic macromolecules and the MWCNTs surface.

The yields of HA:MWCNTs-10, HA:MWCNTs-20, and HA:MWCNTs-30 composites range from 80.28 to 89%, exceeding the yield of the initial HA (70.34%).

These results demonstrate the efficiency of the selected ultrasound-assisted co-precipitation method for the preparation of composite materials based on humic acids and multi-walled carbon nanotubes.

Overall, the results of elemental analysis and conductometric titration show that the incorporation of MWCNTs makes it possible to modify the elemental and functional composition of the composite materials while retaining a significant amount of oxygen-containing functional groups, which is of interest for their further application in sorption technologies [23]. The observed changes in elemental composition and functional-group content indicate that incorporation of MWCNTs does not simply dilute the humic component but also affects the spatial arrangement of humic macromolecules. As a result, the accessibility of oxygen-containing functional groups depends not only on their overall content but also on the degree of dispersion and interfacial interactions within the composite structure.

### 3.2. Influence of Ultrasonic Treatment Time on the Content of Oxygen-Containing Functional Groups (Σ(COOH+OH)) and the Yield of Composite Materials

Table 3 shows the effect of ultrasonic treatment duration on the total content of oxygen-containing functional groups, Σ(COOH+OH), and the yield of HA:MWCNTs composites at different component mass ratios.

At an ultrasonic treatment duration of 15 min, all investigated composites (HA:MWCNTs-10, HA:MWCNTs-20, and HA:MWCNTs-30) exhibit the lowest values of Σ(COOH+OH) (3.00–3.90 mmol/g), while the composite yields range from 75.02 to 85.00%. The obtained results may be associated with an insufficient degree of MWCNTs dispersion and incomplete development of interfacial interactions between the system components during the initial stage of ultrasonic treatment.

An increase in the ultrasonic treatment duration to 30 min is accompanied by an increase in both Σ(COOH+OH) values and composite yields, which reach maximum values for all investigated compositions (3.36–4.45 mmol/g and 80.00–89.28%, respectively). The obtained data indicate that this treatment regime promotes the formation of composite materials with the highest values of the investigated parameters. Higher values of Σ(COOH+OH) may indicate increased accessibility of oxygen-containing functional groups. Within the investigated range of conditions, an ultrasonic treatment duration of 30 min may be considered the most favorable processing regime.

A further increase in ultrasonic treatment duration to 60 min results in a decrease in both Σ(COOH+OH) values and composite yields compared with the 30 min treatment regime. Under these conditions, the total content of oxygen-containing functional groups ranges from 3.13 to 4.11 mmol/g. The obtained results may indicate a decrease in the accessibility of a portion of the functional groups for analytical determination.

The observed changes in Σ(COOH+OH) as a function of ultrasonic treatment duration are likely caused by a combination of factors, including the degree of MWCNTs dispersion, changes in the spatial organization of humic macromolecules, and the characteristics of component interactions within the HA:MWCNTs system. Under moderate ultrasonic treatment, a more uniform distribution of the humic component over the nanotube surface may occur, contributing to increased accessibility of oxygen-containing functional groups.

When the ultrasonic treatment duration is increased to 60 min, the influence of acoustic cavitation effects becomes more pronounced. This may be accompanied by structural rearrangements of the humic phase and changes in the spatial organization of the system components without significant alteration of their chemical composition [31]. Such changes may result in partial shielding of functional groups and a decrease in the measured value of Σ(COOH+OH).

Thus, the obtained results demonstrate that the duration of ultrasonic treatment has a significant influence on both the functional characteristics and the yield of the composite materials. The observed changes may be associated with the dispersion behavior of the components, the accessibility of oxygen-containing functional groups, and the nature of their interfacial interactions.

### 3.3. Analysis of FTIR Spectra of the Initial Compounds and the HA:MWCNTs Composites

**Figure 2:**
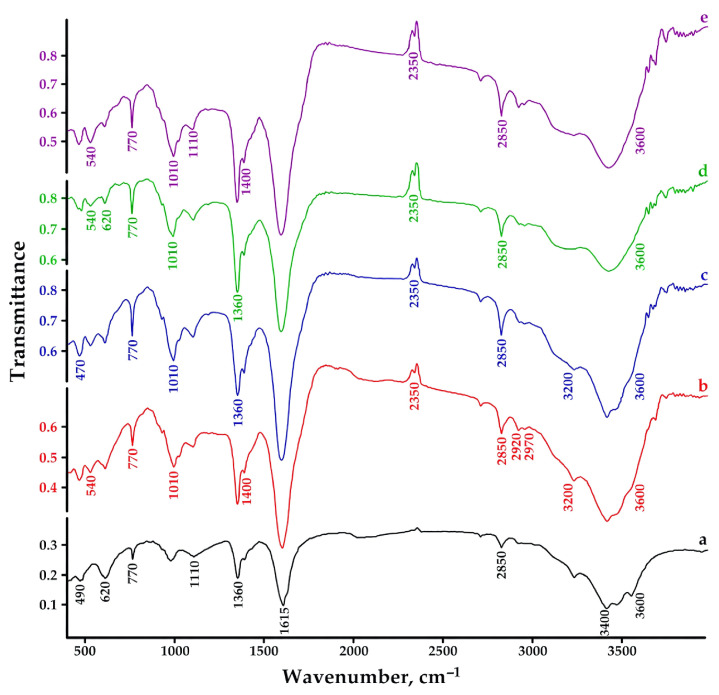
IR spectra of the initial components and synthesized composites: a—multi-walled carbon nanotubes (Sigma Aldrich); b—humic acids; c—composite HA:MWCNTs-20 (US = 15 min); d—composite HA:MWCNTs-20 (US = 30 min); e—composite HA:MWCNTs-20 (US = 60 min).

Figure 2 shows the FTIR spectra of the original multi-walled carbon nanotubes (a), humic acids (b), and HA:MWCNTs-20 composites obtained with different ultrasonic treatment times (c–e).

The IR spectrum of multiwalled carbon nanotubes (MWCNTs) contains characteristic absorption bands typical of graphite-like carbon materials. In the low-frequency region of 400–700 cm^−1^, bands are observed that can be attributed to skeletal vibrations of the carbon framework of MWCNTs, likely related to the curvature of the sp^2^-hybridized carbon network and the presence of structural defects. A weak band near 770 cm^−1^ can be attributed to defect-related vibrations in the graphitic structure and possibly to out-of-plane deformation modes of aromatic C–H bonds. The band at 1615 cm^−1^ may be associated with stretching vibrations of C=C bonds in the sp^2^-hybridized carbon network [32].

The presence of oxygen-containing groups on the MWCNTs surface is confirmed by bands in the 1000–1300 cm^−1^ region, particularly the band near 1110 cm^−1^, which may be associated with C–O vibrational modes of various oxidized surface functionalities. The band at ~1360 cm^−1^ may originate from defect-induced vibrations and/or symmetric stretching vibrations of carboxylate groups. Absorption bands in the 3400–3600 cm^−1^ region are attributed to O–H stretching modes of hydroxyl and carboxyl groups, including adsorbed water on the nanotube surface.

The IR spectra of humic acid (HA) and HA:MWCNTs composites exhibit absorption bands characteristic of aromatic and aliphatic structures, as well as oxygen-containing functional groups. The spectrum contains a band at ~1010 cm^−1^, which may be attributed to C–O stretching vibrations in phenolic, ester, or C–O–C fragments. A band in the 1700–1720 cm^−1^ region can be assigned to C=O stretching vibrations of carboxylic and carbonyl groups. The band at about 1400 cm^−1^ may reflect symmetric vibrations of carboxylate groups. Weak absorption observed near 2350–2400 cm^−1^ is attributed to atmospheric CO_2_. The bands in the 2850–2970 cm^−1^ region correspond to symmetric and asymmetric C–H stretching vibrations of aliphatic methyl and methylene groups. A broad band in the 3200–3600 cm^−1^ range reflects O–H stretching modes of hydroxyl groups, including phenols, alcohols, and adsorbed water.

Compared to pristine HA and MWCNTs, the FTIR spectra of the HA:MWCNTs composites exhibit noticeable changes in both band intensity and position. In particular, a slight shift and broadening of the O–H stretching band (3200–3600 cm^−1^) suggest enhanced hydrogen-bonding interactions between functional groups of humic acid and oxidized sites on the MWCNTs surface. Additionally, variations in the relative intensity of the bands in the ~1710–1600 cm^−1^ and 1000–1300 cm^−1^ regions indicate possible interactions involving carboxyl and phenolic groups of humic acid with the carbon nanotube surface. These spectral changes become more pronounced with increasing ultrasonic treatment time, suggesting stronger interfacial interactions and possibly improved dispersion of MWCNTs within the humic acid matrix. These observations are in good agreement with the conductometric titration results, which showed the highest accessibility of oxygen-containing functional groups for the composite prepared at US = 30 min. The combined FTIR and conductometric data therefore suggest that moderate ultrasonic treatment promotes more efficient interaction between humic acid macromolecules and MWCNTs without causing excessive structural rearrangement.

### 3.4. Thermogravimetric Analysis of Composite Materials

**Figure 3:**
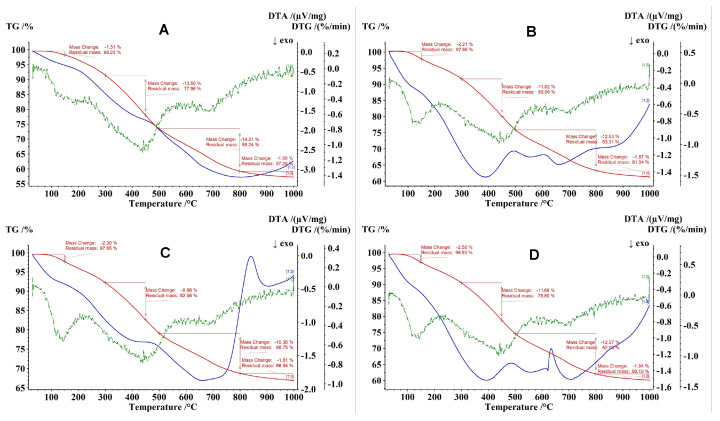
Thermogravimetric curves of samples: (**A**) HA; (**B**) composite HA:MWCNTs-20 (US = 15 min); (**C**) composite HA:MWCNTs-20 (US = 30 min); (**D**) composite HA:MWCNTs-20 (US = 60 min).

Figure 3 presents the TGA/DTG curves of the initial HA (A) and HA:MWCNTs-20 composites obtained at different ultrasonic treatment durations (B—15 min, C—30 min, D—60 min) over the temperature range up to 1000 °C. Analysis of the TGA/DTG curves shows that all investigated samples exhibit a multistage thermal decomposition behavior. In the temperature range of 120–150 °C, the removal of physically adsorbed moisture is observed. The main weight losses occur within the 300–450 °C range and are associated with the thermal decomposition of oxygen-containing functional groups of the humic component, including carboxyl and phenolic groups, as well as the destruction of less thermally stable organic structures. Within the 500–800 °C range, a further decrease in sample mass is observed, caused by the thermal transformation of more condensed aromatic fragments and structural rearrangement of the carbon matrix. In the 800–1000 °C region, mass changes are insignificant, indicating the preservation of a thermally stable carbonaceous residue and the completion of the main thermal degradation processes.

For the initial HA (Figure 3A), the total weight losses in the temperature intervals 120–150, 300–450, 500–800, and 800–1000 °C are 1.31, 13.50, 14.21, and 2.00%, respectively. The residual mass at 1000 °C is 57.26%, indicating the presence of both thermolabile oxygen-containing functional groups and more thermally stable carbon fragments within the HA structure.

Compared with the initial HA, the HA:MWCNTs-20 composites are characterized by a higher residual mass at 1000 °C. For samples obtained at ultrasonic treatment durations of 15, 30, and 60 min, this parameter equals 61.34, 66.94, and 60.10%, respectively. The increase in residual mass may be associated with the presence of the thermally stable carbon phase of MWCNTs and changes in the structural organization of the composites.

The highest residual mass is observed for the composite obtained at US = 30 min (Figure 3C). This sample is also characterized by the lowest weight losses within the 300–450 °C (9.66%) and 500–800 °C (10.36%) temperature ranges compared with the composites obtained at US = 15 min (Figure 3B) and US = 60 min (Figure 3D). The obtained results may indicate a higher thermal stability of the composite formed at this ultrasonic treatment duration.

For the composites obtained at US = 15 min and US = 60 min, the weight losses within the indicated temperature ranges are higher and amount to 11.62–11.68% and 12.53–12.57%, respectively. This may be associated with differences in the structural organization of the composites and the accessibility of oxygen-containing functional groups.

Comparison of the TGA/DTG results with the data on the content of oxygen-containing functional groups, Σ(COOH+OH), reveals a relationship between thermal stability and the structural-functional characteristics of the HA:MWCNTs-20 composites. The composite obtained at US = 30 min is characterized by both the highest residual mass and the highest content of accessible oxygen-containing functional groups among the investigated samples.

The obtained data suggest that ultrasonic treatment duration has a significant influence on the formation of the HA:MWCNTs composite structure. Within the investigated range of conditions, an ultrasonic treatment duration of 30 min provides the most favorable combination of thermal stability and preservation of functional groups. At shorter treatment durations, incomplete dispersion of the system components may occur, whereas prolonged ultrasonic treatment may be accompanied by structural rearrangements of the humic phase and changes in the accessibility of functional centers. The obtained results are consistent with the conductometric titration data and confirm the significant influence of ultrasonic treatment conditions on the properties of HA:MWCNTs composite materials. The improved thermal stability observed for the composite prepared at US = 30 min is likely associated with stronger interfacial interactions between the humic matrix and well-dispersed MWCNTs, which restrict thermal degradation of the organic phase and contribute to the formation of a more stable composite structure.

### 3.5. Investigation of the Surface Morphology of Composite Materials by SEM

**Figure 4:**
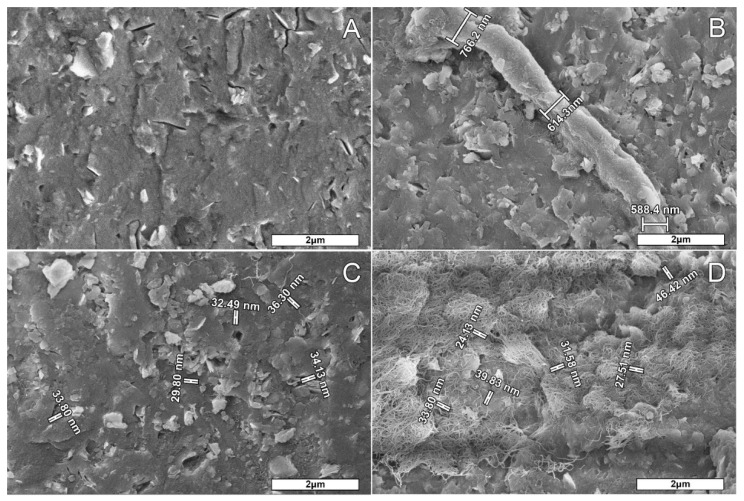
Electron microscopic images: (**A**) HA; (**B**) composite HA:MWCNTs-20 (US = 15 min); (**C**) composite HA:MWCNTs-20 (US = 30 min); (**D**) composite HA:MWCNTs-20 (US = 60 min).

Electron microscopic images of the initial HA and HA:MWCNTs-20 composites obtained at different ultrasonic treatment durations are presented in Figure 4A–D. Analysis of the SEM images showed that the morphology of the initial HA and the HA:MWCNTs-20 composites strongly depends on the duration of ultrasonic treatment. The initial HA (Figure 4A) is characterized by a relatively dense surface containing individual plate-like structural fragments, local microcracks, and a poorly developed porous structure.

For the HA:MWCNTs-20 composite obtained at US = 15 min (Figure 4B), large aggregated regions with a non-uniform distribution of fibrous MWCNTs elements are observed. The surface morphology is characterized by pronounced heterogeneity, while some areas exhibit densely packed structural formations. The obtained results may indicate an insufficient degree of MWCNTs dispersion within the humic matrix and the preservation of aggregated regions in the composite structure. The presence of such morphological heterogeneities may be associated with reduced accessibility of oxygen-containing functional groups, which is consistent with the decrease in the Σ(COOH+OH) value to 3.9 mmol/g compared with the initial HA (5.0 mmol/g).

Increasing the ultrasonic treatment duration to 30 min (Figure 4C) results in the formation of a more homogeneous composite morphology and a more uniform distribution of fibrous MWCNTs elements. The characteristic dimensions of individual fibrous structures are approximately 21.39–36.30 nm. The obtained results are consistent with the data on the total content of oxygen-containing functional groups: this sample exhibits the highest Σ(COOH+OH) value among the investigated composites, equal to 4.45 mmol/g. Furthermore, according to the TGA results, this sample is characterized by the highest residual mass at 1000 °C, which may indicate enhanced thermal stability of the composite. The combination of these results suggests that an ultrasonic treatment duration of 30 min provides the most favorable balance between MWCNTs dispersion and preservation of the functional properties of the humic component.

For the sample obtained at US = 60 min (Figure 4D), a well-developed fibrous network morphology with a non-uniform distribution of structural elements throughout the composite volume is observed, which is consistent with literature data reported for similar systems [24]. Interwoven fibrous MWCNTs formations forming regions of increased structural density can be observed. The characteristic dimensions of individual fibrous elements are approximately 24.13–46.42 nm. At the same time, local regions of secondary MWCNTs aggregation and structural heterogeneity are detected, which may be associated with changes in the nature of component interactions during prolonged ultrasonic treatment. The obtained morphological data are consistent with the decrease in Σ(COOH+OH) to 4.11 mmol/g and a slight reduction in thermal stability compared with the sample treated for 30 min.

Thus, the results of SEM analysis, determination of Σ(COOH+OH), and TGA demonstrate that the duration of ultrasonic treatment has a significant influence on the morphology and properties of HA:MWCNTs composites. Among the investigated samples, the most favorable combination of morphological, functional, and thermal characteristics was observed for the composite obtained at US = 30 min. The obtained results suggest that this treatment regime promotes a more uniform distribution of the components and preserves the accessibility of oxygen-containing functional groups within the composite structure. The improved dispersion observed after 30 min of ultrasonic treatment is also consistent with the adsorption results discussed below, indicating that a more homogeneous composite structure provides better accessibility of active adsorption sites for phenol molecules.

### 3.6. Adsorption Properties of HA:MWCNT-20 Composites Depending on Ultrasonic Treatment Duration

**Figure 5:**
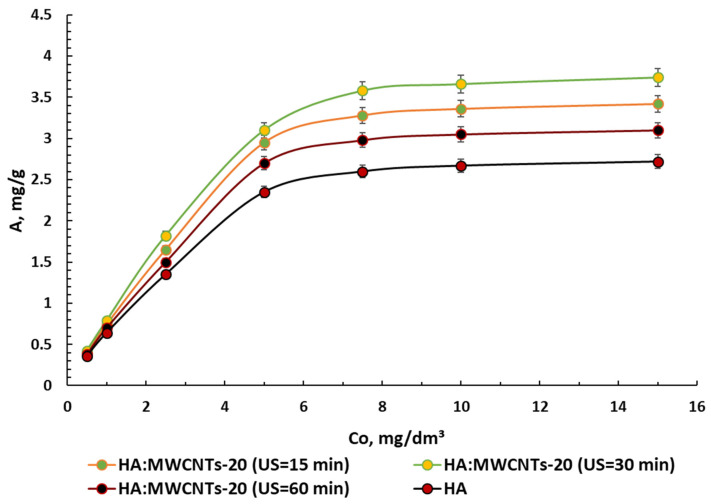
Adsorption isotherms of phenol on HA and HA:MWCNTs-20 composites as a function of concentration.

Figure 5 presents the phenol adsorption isotherms obtained for the initial HA and the HA:MWCNTs-20 composite synthesized at different ultrasonic treatment durations (15, 30, and 60 min). The HA:MWCNTs-20 composite was selected for phenol adsorption studies based on the preliminary comparative evaluation of the synthesized compositions. Among the investigated HA:MWCNTs ratios, this sample demonstrated the most favorable combination of product yield, elemental composition, and accessible oxygen-containing functional groups, which are important factors for interaction with phenol molecules. The HA:MWCNTs-10 composite contained a lower fraction of the carbon nanotube component, whereas HA:MWCNTs-30 showed a decrease in Σ(COOH+OH), which may be associated with partial shielding of humic functional groups at higher MWCNTs content. Therefore, HA:MWCNTs-20 was selected as the most promising composition for detailed adsorption studies and for evaluating the effect of ultrasonic treatment duration.

All obtained isotherms exhibit a characteristic convex shape with the appearance of a plateau as the equilibrium phenol concentration increases, indicating gradual saturation of the active adsorption sites of the sorbent. According to the Giles classification, the obtained dependences belong to L-type isotherms, which are characteristic of systems exhibiting pronounced adsorbate–adsorbent interactions. The observed shape of the isotherms also suggests that the adsorption process can be described using the Langmuir model.

Comparative analysis shows that modification of HA with MWCNTs enhances the adsorption capacity of the material toward phenol. Among the investigated samples, the highest adsorption capacity was observed for the HA:MWCNTs-20 composite obtained at US = 30 min. For this sample, the sorption capacity reaches 3.7–3.8 mg/g, exceeding the corresponding values for both the initial HA and the composites obtained at ultrasonic treatment durations of 15 and 60 min.

The composite synthesized at US = 15 min exhibits a somewhat lower adsorption capacity, which may be associated with an insufficient degree of MWCNTs dispersion and a less uniform distribution of nanotubes within the humic matrix, thereby limiting the accessibility of some adsorption-active sites. Increasing the ultrasonic treatment duration to 60 min results in a decrease in sorption capacity compared with the sample obtained at US = 30 min. The observed effect may be attributed to changes in the structural organization of the composite and a decrease in the accessibility of some oxygen-containing functional groups involved in interactions with phenol molecules.

The initial HA is characterized by the lowest adsorption capacity (2.6–2.7 mg/g), indicating the positive effect of MWCNTs incorporation on the sorption properties of the material. The enhanced adsorption performance of the composites may be associated with changes in surface structure and an expanded spectrum of interactions with phenol molecules, including π–π interactions, hydrogen bonding, and donor–acceptor interactions. The superior adsorption performance of the composite prepared at US = 30 min can therefore be explained by the combined effect of improved nanotube dispersion, higher accessibility of oxygen-containing functional groups, and enhanced structural homogeneity, as confirmed by the FTIR, SEM, conductometric titration, and thermogravimetric analyses.

The obtained results demonstrate that ultrasonic treatment duration has a significant influence on the adsorption properties of HA:MWCNTs composites. Among the investigated samples, the highest adsorption capacity was observed for the HA:MWCNTs-20 composite obtained at US = 30 min. The maximum sorption capacity of this composite is 3.7–3.8 mg/g, exceeding the values obtained for the initial HA and the composites synthesized at ultrasonic treatment durations of 15 and 60 min.

It should be noted that the obtained adsorption capacities were determined within the initial phenol concentration range of 0.5–15 mg/dm^3^, which was limited by the operating range of the analytical equipment used. The upper limit of the working range was 20 mg/dm^3^, which restricted the possibility of studying adsorption at higher phenol concentrations.

Therefore, the obtained results should not be directly compared with the sorption capacities of highly porous carbon adsorbents investigated at substantially higher phenol concentrations. According to literature data, activated carbons, multi-walled carbon nanotubes, and MWCNTs-based materials are capable of exhibiting considerably higher phenol adsorption capacities [1,6,24,33,34]. The observed differences may be attributed to differences in the structure and surface chemistry of the sorbents, as well as variations in experimental conditions, including the phenol concentration range and adsorption parameters.

Thus, the HA:MWCNTs-20 composite should be considered a functional humic-carbon material that is promising for the removal of phenol from aqueous solutions at low contaminant concentrations. The obtained results indicate that the effectiveness of this material is determined by the combination of the humic matrix and MWCNTs, as well as by the possibility of regulating the accessibility of functional groups through variation of the ultrasonic treatment duration.

**Figure 6:**
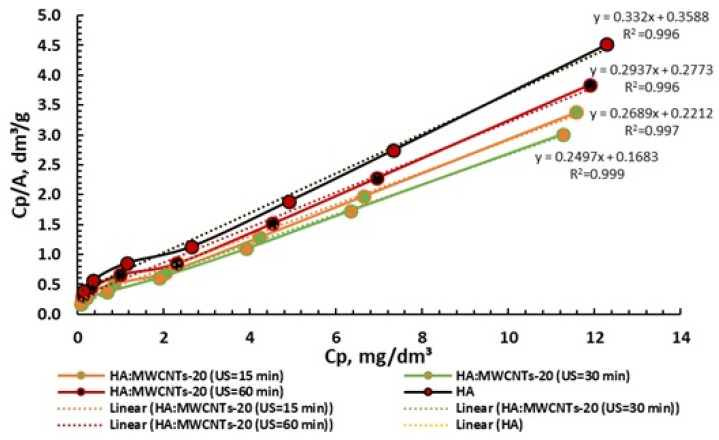
Isotherm of phenol sorption on HA and HA:MWCNTs-20 composites obtained with different durations of ultrasonic treatment in the coordinates of the linear form of the Langmuir equation.

Figure 6 presents the experimental dependence of C_eq_/A versus the equilibrium phenol concentration C_eq_, plotted according to the linear form of the Langmuir equation.

For all investigated samples, a nearly linear relationship is observed in the coordinates of the Langmuir equation, indicating good applicability of this model for describing phenol adsorption on the investigated materials. The obtained dependence indicates the presence of a limited number of active adsorption sites and gradual filling of the surface by the adsorbate. At the same time, agreement with the Langmuir model should not be considered sufficient evidence for a strictly monolayer adsorption mechanism, since the process may also involve surface heterogeneity and intermolecular interactions between adsorbed molecules.

Comparative analysis of the slopes of the linear plots (1/A_max_) demonstrates differences in the maximum sorption capacities of the investigated materials. The smallest slope, corresponding to the highest limiting adsorption capacity, is observed for the HA:MWCNTs-20 composite obtained at US = 30 min. This observation is consistent with the results obtained from the analysis of the experimental adsorption isotherms and indicates the highest adsorption performance of this sample toward phenol.

The initial HA exhibits a lower adsorption capacity compared with the composite materials. Modification of HA with multi-walled carbon nanotubes may contribute to increased accessibility of active sites and improvement of the sorption properties of the composites.

High correlation coefficients (r = 0.996–0.999) indicate excellent agreement between the experimental data and the Langmuir model and demonstrate its high applicability for describing phenol adsorption within the investigated concentration range. The obtained results suggest that adsorption proceeds predominantly on relatively homogeneous active sites of the surface. However, agreement with the Langmuir model should not be regarded as direct proof of a strictly monolayer adsorption mechanism.

**Figure 7:**
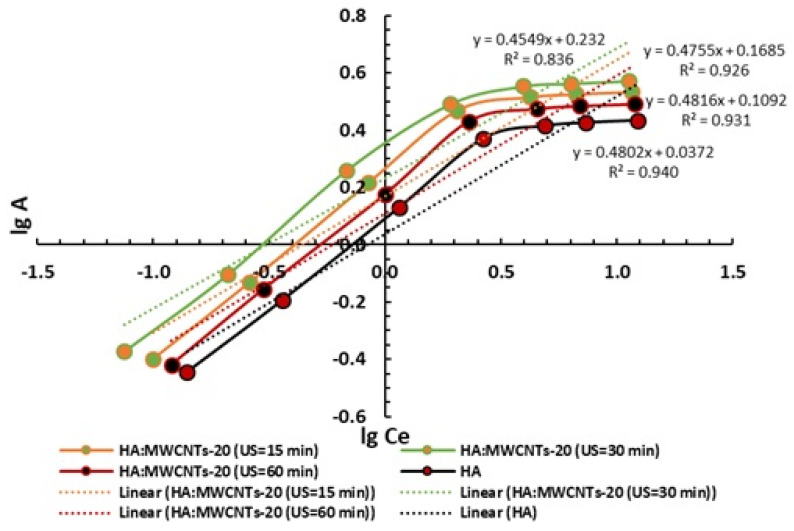
Isotherm of phenol sorption on HA and HA:MWCNTs-20 composites obtained with different durations of ultrasonic treatment in the coordinates of the linear form of the Freundlich equation.

Figure 7 presents the phenol sorption isotherms on HA and HA:MWCNTs-20 composites plotted in the coordinates of the linear form of the Freundlich equation.

For all investigated samples, the Freundlich model provides a satisfactory description of the experimental data only within a limited concentration range. The correlation coefficients (r = 0.836–0.940) are lower than those obtained for the Langmuir model, indicating a less accurate agreement between the Freundlich model and the experimental data. The lower correlation coefficients and the observed deviations from linearity indicate the limited applicability of the Freundlich model for describing phenol adsorption within the investigated concentration range. Therefore, the results of the Freundlich approximation should be considered primarily as an additional characteristic of the energetic and structural heterogeneity of the adsorbent surface [1].

**Figure 8:**
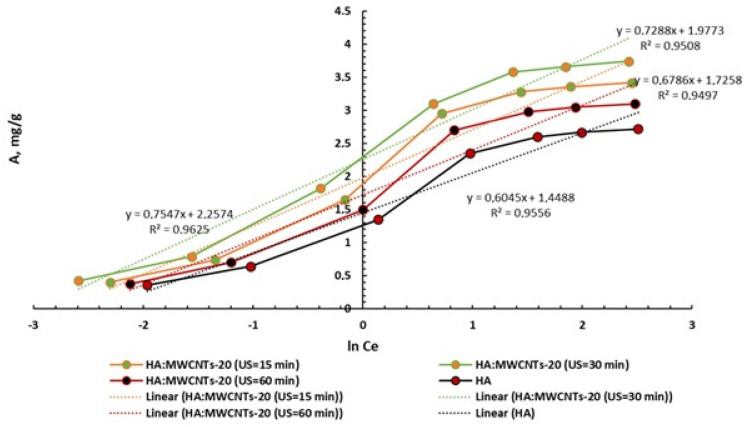
Temkin adsorption isotherms for phenol adsorption on HA and HA:MWCNT-20 composites obtained at different ultrasonic treatment times.

Figure 8 presents the phenol sorption isotherms on HA and HA:MWCNTs-20 composites obtained at different ultrasonic treatment durations in the coordinates of the linear form of the Temkin equation.

For additional evaluation of adsorbate–adsorbent interactions and the energetic heterogeneity of the surface, the experimental data were analyzed using the Temkin model. The linear relationships between A and ln C_eq_ obtained for all investigated samples are characterized by correlation coefficients in the range of r = 0.949–0.962, indicating satisfactory agreement between the experimental data and the Temkin model. The obtained results suggest a possible contribution of adsorbate–adsorbent interactions and a change in adsorption energy as the sorbent surface becomes progressively occupied.

Table 4, Table 5 and Table 6 present the results of the linear approximation of the experimental data for phenol adsorption on HA and HA:MWCNTs-20 composites obtained at different ultrasonic treatment durations, as well as the calculated parameters of the Langmuir, Freundlich, and Temkin models.

Comparative analysis of the linear approximation parameters and correlation coefficients presented in Table 4, Table 5 and Table 6 demonstrates that the Langmuir model provides a better fit to the experimental data than the Freundlich and Temkin models. For all investigated samples, the correlation coefficients for the Langmuir model are in the range of r = 0.996–0.999, whereas for the Freundlich and Temkin models they fall within r = 0.836–0.940 and r = 0.950–0.963, respectively. These results indicate the higher applicability of the Langmuir model for describing the phenol adsorption process within the investigated concentration range.

The highest value of the limiting specific adsorption according to the Langmuir model was observed for the HA:MWCNTs-20 composite obtained at US = 30 min (A_∞_ = 4.0051 mg/g). This is accompanied by the maximum value of the adsorption equilibrium constant (K_L_ = 1.4833 dm^3^/mg). The obtained results indicate a higher affinity of the surface of this composite toward phenol compared with the other investigated samples. For the composites obtained at US = 15 min and US = 60 min, the corresponding A_∞_ values are 3.7185 and 3.0490 mg/g, respectively, whereas for the initial HA, this parameter is 3.0124 mg/g.

The Freundlich model provides a satisfactory description of the experimental data only within a limited concentration range. At the same time, the parameters of the Freundlich model may be used as an additional characteristic of the energetic and structural heterogeneity of the adsorbent surface.

Compared with the Freundlich model, the Temkin model is characterized by higher correlation coefficients, which may indicate a significant contribution of adsorbate–adsorbent interactions and changes in adsorption energy as the surface becomes progressively occupied. Nevertheless, the highest correlation coefficients are observed for the Langmuir model, indicating its superior applicability for describing phenol adsorption on the investigated composite materials [35].

**Figure 9:**
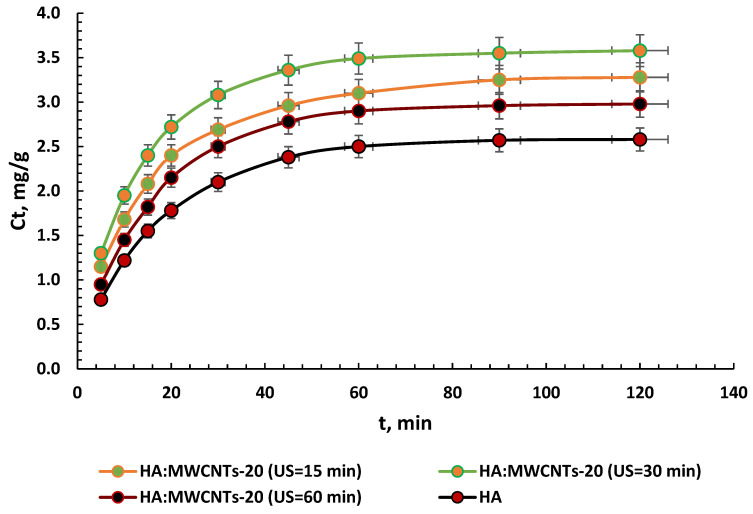
Adsorption isotherms of phenol on HA and HA:MWCNTs-20 composites as a function of time.

Figure 9 presents the time dependence of phenol adsorption on HA and HA:MWCNTs-20 composites obtained at different ultrasonic treatment durations.

Based on the experimental data, the parameters of three kinetic models were calculated and the corresponding correlation coefficients were determined, as presented in Table 7.

Analysis of the kinetic data showed that phenol adsorption on HA and HA:MWCNTs-20 composites obtained at different ultrasonic treatment durations is most satisfactorily described by the pseudo-second-order model. For all investigated samples, the correlation coefficients of this model are the highest and fall within the range of r = 0.9979–0.9996, whereas for the pseudo-first-order and Weber-Morris models the corresponding values are r = 0.9593–0.9866 and r = 0.7990–0.8461, respectively.

The highest value of the pseudo-second-order rate constant (k_2_ = 0.0294) was observed for the composite obtained at US = 30 min, indicating the best agreement between the experimental data and this kinetic model. For the composites obtained at ultrasonic treatment durations of 15 min and 60 min, the corresponding k_2_ values are 0.0267 and 0.0273, respectively, while for the initial HA, this parameter equals 0.0281.

Thus, the obtained results demonstrate that modification of HA with MWCNTs leads to an increase in the adsorption capacity of the materials toward phenol. The results also indicate a significant influence of ultrasonic treatment duration on the formation of the structure and sorption properties of HA:MWCNTs-20 composites. It was established that the HA:MWCNTs-20 composite obtained at US = 30 min possesses the highest sorption characteristics among the investigated samples.

The obtained results are in agreement with the literature data, according to which the pseudo-second-order model provides higher correlation coefficients than the pseudo-first-order model and therefore more satisfactorily describes the kinetics of phenol adsorption. The Weber-Morris plots do not pass through the origin, indicating that intraparticle diffusion contributes to the adsorption process but is not the sole rate-limiting step [33,34].

## 4. Conclusions

In this study, HA:MWCNTs composites were synthesized by an ultrasound-assisted co-precipitation method, and the influence of ultrasonic treatment duration on their structure and adsorption properties toward phenol was investigated. A combination of physicochemical characterization methods confirmed the formation of composite materials with interfacial interactions between the components. The values of Σ(COOH+OH) for the synthesized composites were found to range from 3.00 to 4.45 mmol/g, with the maximum value observed for the HA:MWCNTs-20 sample. FTIR spectroscopy data indicate the participation of hydroxyl and carboxyl groups of humic acids in interfacial physicochemical interactions with the MWCNTs surface.

Comparison of the results obtained by SEM, TGA/DTA, and determination of Σ(COOH+OH) demonstrated the significant influence of ultrasonic treatment duration on the structure and properties of HA:MWCNTs composites. The most favorable combination of morphological, functional, thermal, and sorption characteristics was observed for the HA:MWCNTs-20 composite obtained at an ultrasonic treatment duration of 30 min, for which the maximum values of limiting adsorption capacity and adsorption equilibrium constant were established.

Analysis of the experimental data showed that the phenol adsorption process is most satisfactorily described by the Langmuir model, whereas the kinetic data are best fitted by the pseudo-second-order model.

The obtained results indicate that HA:MWCNTs composites are promising sorption materials for the removal of phenol from aqueous solutions and that optimization of ultrasonic treatment conditions is an effective approach for controlling their structural and functional properties.

## Figures and Tables

**Table 1:** Comparison of the present study with previously reported humic acid/carbon nanomaterial-based composites.

Research Aspect	Current State of Knowledge	Contribution of This Work
Preparation of HA/carbon composites	Various preparation methods have been reported	Ultrasound-assisted co-precipitation under controlled synthesis conditions
Effect of ultrasonic treatment	Insufficiently investigated	Systematic study of treatment duration
Structural characterization	Usually limited to individual techniques	Combined elemental analysis, FTIR, SEM, TGA/DTA and functional-group determination
Adsorption studies	Mainly heavy metals and dyes	Phenol adsorption with isotherm and kinetic analysis

**Table 2:** Elemental composition and functional characteristics of the initial components and composites HA:MWCNTs.

Sample	Ratio	C, wt.%	H, wt.%	O, wt.%	Σ(COOH+OH), mmol/g	Yield, %
HA	–	47.34 ± 0.2	4.35 ± 0.1	48.31 ± 0.2	5.00 ± 0.6	70.34
HA:MWCNTs-10	90:10	51.12 ± 0.2	4.05 ± 0.1	44.83 ± 0.2	3.83 ± 0.7	85.01
HA:MWCNTs-20	80:20	55.61 ± 0.2	3.67 ± 0.1	40.72 ± 0.2	4.45 ± 0.7	89.45
HA:MWCNTs-30	70:30	59.15 ± 0.2	3.25 ± 0.1	37.60 ± 0.2	3.30 ± 0.6	80.28

**Table 3:** Effect of ultrasonic treatment time on the content of Σ(COOH+OH) and the yield of HA:MWCNTs.

Sample	US, min	Σ(COOH+OH), mmol/g	Yield, %
HA:MWCNTs-10	15	3.35 ± 0.7	81.38
	30	3.94 ± 0.6	86.25
	60	3.40 ± 0.6	83.05
HA:MWCNTs-20	15	3.90 ± 0.7	85.00
	30	4.45 ± 0.5	89.28
	60	4.11 ± 0.7	86.00
HA:MWCNTs-30	15	3.00 ± 0.5	75.02
	30	3.36 ± 0.6	80.00
	60	3.13 ± 0.7	77.35

**Table 4:** Linear approximation results and parameters of the Langmuir equations for phenol adsorption.

Sample	k	b	Limiting Specific Adsorption, A_∞_, mg/g	Adsorption Equilibrium Constant, K_L_, dm^3^/mg	r
HA	0.332	0.359	3.0124	0.9252	0.996
HA:MWCNTs-20 US = 15 min	0.269	0.221	3.7185	1.2155	0.997
HA:MWCNTs-20 US = 30 min	0.250	0.168	4.0051	1.4833	0.999
HA:MWCNTs-20 US = 60 min	0.294	0.277	3.0490	1.0593	0.996

**Table 5:** Linear approximation results and parameters of the Freundlich equations for phenol adsorption.

Sample	k	b	K_Fr_	n	r
HA	0.480	0.037	0.9597	2.0824	0.940
HA:MWCNTs-20 US = 15 min	0.476	0.169	0.8315	2.1030	0.926
HA:MWCNTs-20 US = 30 min	0.455	0.125	0.8771	2.1983	0.836
HA:MWCNTs-20 US = 60 min	0.482	0.109	0.8860	2.0824	0.931

**Table 6:** Linear approximation results and parameters of the Temkin equations for phenol adsorption.

Sample	k	b	r
HA	0.605	1.449	0.956
HA:MWCNTs-20 US = 15 min	0.679	1.726	0.950
HA:MWCNTs-20 US = 30 min	0.755	2.257	0.963
HA:MWCNTs-20 US = 60 min	0.729	1.977	0.951

**Table 7:** Kinetic equations of phenol adsorption on HA and composites obtained with different durations of ultrasonic treatment and correlation coefficients (r).

**Pseudo-Order of Reaction**	**Adsorption of Phenol by HA**		
First	lnC = −0.042·t + 0.593	r = 0.9707	k_1_ = 0.0418
Second	1/C = 0.345·t+ 4.227	r = 0.9979	k_2_ = 0.0281
Weber-Morris	C_t_ = 0.202·t^1/2^ + 6.774	r = 0.8396	k_3_ = −0.2019
**Pseudo-order of reaction**	**Adsorption of phenol by composite** **HA:MWCNTs-20 (US = 15 min)**		
First	lnC = −0.061·t + 1.279	r = 0.9593	k_1_ = −0.0608
Second	1/C = 0.278·t + 2.893	r = 0.9996	k_2_ = 0.0267
Weber-Morris	C_t_ = 0.233·t^1/2^ + 6.390	r = 0.8461	k_3_ = −0.2328
**Pseudo-order of reaction**	**Adsorption of phenol by composite** **HA:MWCNTs-20 (US = 30 min)**		
First	lnC = −0.062·t + 1.188	r = 0.9741	k_1_ = 0.0617
Second	1/C = 0.257·t + 2.248	r = 0.9990	k_2_ = 0.0294
Weber-Morris	C_t_ = 0.244·t^1/2^ + 6.139	r = 0.7990	k_3_ = −0.2436
**Pseudo-order of reaction**	**Adsorption of phenol by composite** **HA:MWCNTs-20 (US = 60 min)**		
First	lnC = 0.063·t + 1.131	r = 0.9866	k_1_ = 0.0627
Second	1/C = 0.302·t + 3.340	r = 0.9979	k_2_ = 0.0273
Weber-Morris	C_t_ = 0.225·t^1/2^ + 6.579	r = 0.8192	k_3_ = −0.2255

## Data Availability

The original contributions presented in this study are included in the article. Further inquiries can be directed to the corresponding author.

## Abbreviations

The following abbreviations are used in this manuscript:

HA	Humic acids
IR	Infrared
MWCNTs	Multi-walled carbon nanotubes
SEM	Scanning electron microscopy
TGA	Thermogravimetric analysis
